# Developmental psychopathology: Attention Deficit Hyperactivity Disorder (ADHD)

**DOI:** 10.1186/1471-244X-9-58

**Published:** 2009-09-17

**Authors:** Sören Schmidt, Franz Petermann

**Affiliations:** 1 Centre for Clinical Psychology and Rehabilitation, University of Bremen, Bremen, Germany

## Abstract

**Background:**

Attention Deficit/Hyperactivity Disorder (ADHD), formerly regarded as a typical childhood disorder, is now known as a developmental disorder persisting over the lifespan. Starting in preschool-age, symptoms vary depending on the age group affected.

**Method:**

According to the variability of ADHD-symptoms and the heterogeneity of comorbid psychiatric disorders, a broad review of recent studies was performed. These findings were summarized in a developmental psychopathological model, documenting relevant facts on a timeline.

**Results:**

Based on a genetic disposition and a neuropsychological deregulation, there is evidence for factors which persist across the lifespan, change age-dependently, or show validity in a specific developmental phase. Qualitative changes can be found for children in preschool-age and adults.

**Conclusion:**

These differences have implications for clinical practice as they can be used for prevention, diagnostic proceedings, and therapeutic intervention as well as for planning future studies. The present article is a translated and modified version of the German article "Entwicklungspsychopathologie der ADHS", published in *Zeitschrift für Psychiatrie, Psychologie und Psychotherapie*, 56, 2008, S. 265-274.

## Background

Formerly regarded as a typical disorder in childhood and adolescence, Attention-Deficit/Hyperactivity-Disorder (ADHD) is increasingly discussed as a serious psychiatric disorder in adulthood [1-6]. Thereby ADHD shows high heterogeneity and comorbidity with other psychiatric disorders (e.g. Borderline Personality Disorder) [7-10].

Considering the developmental course of ADHD over the lifespan, evidence exists that increasing age has an influence on the heterogeneity of ADHD-symptoms and associated impairments. Regarding the fact that in many cases hyperactivity is not primarily associated with the typical deficits of adult ADHD, a developmental change is discussed [10]. Symptoms such as hyperactivity are not the core symptoms in adulthood. Whereas children primarily have problems in school, in adulthood different areas of functioning are negatively affected (e.g. partnership, work, social contact).

Today ADHD-specific-studies, instruments for psychological assessment, and intervention programs are available mainly for the treatment of children and adolescents. However, in consideration of relevant publications in the last five years, there is strong evidence for the occurrence of ADHD-related symptoms in preschool age and adulthood which has implications for the development of assessment procedures and intervention programs [11-13]. From a developmental psychopathological point of view, the preschool age in particular is a matter of clinical interest since early prognostic factors can alleviate a psychological intervention and consequently prevent a pathological development [14].

### ADHD - a lifespan disorder

Today ADHD is considered a multifactorial psychiatric disorder, based on genetic predisposition and neurobiological deregulation. These lead to a neuropsychological inhibitory deficit which contributes to the specific impairments typical for ADHD [15-18].

#### Genetics

There is strong evidence for a genetic disposition as a basis for ADHD. Numerous twin-studies exist that indicate a familiar interrelation [19]. Faraone et al. analyzed 20 extant twin-studies and estimated the mean heritability of ADHD up to 76% [20]. Molecular genetic studies focus on mutations in the DNA-sequences, which have a negative influence on the proteins of the dopaminergic neurons and lead to a dysfunction. Catecholaminergic genes, in particular the dopamine receptor D4 (DRD4) gene, play an important role. An association between frontal subcortical networks and ADHD has been proven in different studies [15,21]. Other genes discussed in connection with ADHD are the dopamine receptor genes DRD5, DRD2, DRD3, DRD1 as well as the dopamine transporter gene DAT1 [17,19,22].

#### Neuroanatomy and neurobiology

In case of ADHD, the functional impairment of attention performance is regarded as resulting from a dysfunction of frontal-cortical-networks. Many symptoms in ADHD-afflicted persons (e.g. deficits in focused attention, working memory, executive-functions) are comparable with symptoms from patients suffering from frontal lobe damage, which highlights the importance of the frontal cortical networks [4,7]. Numerous studies demonstrated complex processing mechanisms depending on a specific attention dimension [23]. In case of deficits in inhibition and visuospatial working memory, functional impairments of the right inferior frontal gyrus can be observed in lesion studies [24]. The activation seems to be influenced by the noradrenergic system (locus coeruleus) and its projections in the right hemisphere. Furthermore, a regulation of this process through the right prefrontal cortex is assumed, which is supported by findings in related studies that report a regional volume reduction [25]. Ströhle et al. reported deficits of executive and motivational factors in an fMRI-Study, using a paradigm of positive feedback and removal of positive feedback. Deficits where seen in consequence of a decreased activation in the ventral striatum (expectation of positive feedback) and an increased activation in the orbito frontal cortex (answer on the type of feedback). Thereby negative correlations between self-described hyperactivity and impulsivity and the decreased ventral striatal activation could be observed [26]. Regarding the effect of ADHD on all brain regions, Castellanos et al. performed a neuroimaging study, focusing on developmental trajectories [27]. They found that activity in nearly all brain regions was significantly decreased in children and adolescents with ADHD (about 3%, adjusted). Interestingly, the authors did not find evidence for primarily frontal abnormalities as mentioned above. However, based on a larger analysis of the different brain units, they conclude that these findings cannot be regarded as evidence against the interrelation between the ADHD symptomatology and frontal-striatal networks. Focusing on the fundamental developmental growth curve of the different brain regions, the authors did not find different trajectories for ADHD affected children or adolescents and controls. These findings implicate that no fundamental developmental process can be observed, even if the regional brain volumes of the ADHD-group are smaller compared to controls.

## Method

Regarding the developmental course ADHD seems to become more unspecific in its psychopathological characteristics over time, although it is based upon the same neuropsychological dysfunctions as in childhood and adolescence. This leads to two fundamental questions:

• During which part of the developmental pathway can qualitative changes be observed?

• What are the reasons for these qualitative changes?

To answer both questions, a broad review of recent studies (published between January 1997 and January 2009) was performed. To this end, scientific databases (e.g. PubMed, Sciencedirect, ISI Web of Knowledge, Springerlink) were searched, using the keywords or keyword combinations of *ADHD*, *prevalence*, *preschool*, *childhood*, *adolescence*, *adulthood*, *lifespan*, *comorbidity*, *developmental*, *genetic*, *neuropsychology*, *neurobiology*. Based on the evidence for a genetic/neurobiological deregulation as the basis of the developmental course of ADHD, studies highlighting preschool age, school age/adolescence, and adulthood were selected. In a first step, these findings were described under consideration of actual prevalence rates and the specific developmental course (e.g. frequent comorbid disorders and resulting problems). In a second step, findings were summarized in a developmental model and discussed regarding the above mentioned questions and the specific phenomenology of each age group.

## Results

### ADHD in preschool children

#### Prevalence

Until now, only little representative international data on the prevalence rate of ADHD in preschool children exist. The results of the German Child- and Youth Health Survey [28] can be mentioned as an exemplary study that investigated prevalence rates on ADHD among N = 14.836 children between the ages of 3 and 17 years. According to this study, the prevalence rate among preschool children is around 2%. Former studies in the US reported prevalence rates up to 6% [29,30].

#### Developmental course

In this age group, ADHD symptoms are usually assessed by means of rating scales and behavior observations [31]. Today a consensus is established as to how pathological development differs from normal development. In a Swedish study, 131 children between the ages of 3 and 7 and diagnosed with ADHD were compared to an age-matched control group without ADHD [32]. Out of 12 symptoms assessed, the following were suitable to describe ADHD in preschool children (ADHD-Rating Scale-IV) [33]:

• problems with prolonged maintenance of attention,

• a high distractibility,

• being on the go often,

• excessive running/climbing,

• not adhering to instructions,

• having trouble to sit still.

It is necessary to state that in this age group already, a high comorbidity with other behavioural disorders exists. In the Preschool ADHD Treatment Study (PATS) Posner et al. reported comorbid disorders in seventy percent of ADHD cases, the most frequent being oppositional defiant disorder (52.1%), communication disorders (24.7%) and anxiety disorders (17,7%). These findings underline the need for an early intervention and highlight the early impact ADHD can have on the developmental course over the lifespan [30].

### ADHD among school children and adolescents

#### Prevalence

The prevalence rate of ADHD among school children and adolescents diverges from 3.2% to 15.8%, depending on the classification system used. Prevalence rates between 5% and 7% are reported most often [34,35]. Furthermore, boys are two to four times more likely to be diagnosed with ADHD than girls [36-38].

#### Developmental course

There are numerous different diagnostic instruments for this age group. Questionaires and rating scales are based on external sources (parents, teachers, educators) or self-report, depending on the age group (usually from the age of 11 onwards). For the neuropsychological assessment of attention capacity, computer based instruments are used in which attention regulation (stimulus inhibition, attention division, reaction flexibility) and attention load (alertness, attention endurance, vigilance) are measured. Untreated ADHD constitutes a high risk for a further negative development, which is especially due to frequent comorbid disorders. During adolescence, ADHD can negatively impair more and more areas of functioning [39,40].

Among children with ADHD, in up to 65% of cases oppositional behavior is found [41] and among 23% of cases a comorbid anxity disorder can be observed [42]. Furthermore, ADHD often co-occurs with school problems [43] which, among other things, can be linked to comorbid learning disorders such as dyscalculia [44]. Information of teachers, parents and, from a certain age on, self-report data reveal problems with peers, aggressive behavior and diminished achievement motivation. With increasing age, emotional problems increase, which in many cases result from peer rejection, frequent hassles with teachers, as well as the feeling „of being different“ [45]. Often children and adolescents try to connect to peers who have similar problems which has a further negative impact on the child's/adolescent's functioning (e.g. delinquency) [46]. Moreover, a relationship between risky traffic behavior and ADHD was reported [47]. A very important problem is, however, the issue of increased substance abuse. To explain this link, a very complex causation model can be presumed. The ADHD itself (reduced ability to suppress stimuli, impulsivity), the influence of the social environment (peer group, family environment) as well as the physical and cognitive appraisal of the consumption itself (self medication) may play a role [48,49]. To determine predictors for substance abuse, in the last 3 years alone numerous prospective studies were conducted. For nicotine consumption of adolescents and adults with ADHD, a relationship between behavior problems and subsequent tobacco consumption was reported [50]. In the follow-up study of Burke and colleagues a predictive relationship between inattentiveness and tobacco consumption in youth as well as daily tobacco consumption in adulthood was found [39]. A further prospective study detected a strong association between the presence of hyperactivity (at the age of 11 years) and the first consumption of nicotine and other substances at the age of 14 [40]. For cannabis, a prospective birth cohort study (N = 1265; 0-25 age) found an association between early cannabis consumption and ADHD in adulthood, which was moderated by the consumption of other substances [51]. It becomes apparent that the relation of ADHD and the abuse of different substances play a determining role in the transition to adulthood, which makes preventive steps necessary.

### ADHD in adulthood

#### Prevalence

For the transition to adulthood, studies document the persistence of behavioural problems in 40-60% of the cases. Prevalence rates between 1-7.3% were found [9,10,52-54]. While in childhood salient gender differences exist, in adulthood these cannot be found [55].

#### Developmental course

The developmental course is extremely heterogeneous, which, among other things, results from the varied comorbid disorders. Different studies report a strong relationship, particularly with substance abuse, affective disorders, antisocial and borderline personality disorder. The reasons for these overlaps are manifold and are related to the similarity of the neurobiological processing mechanisms of the different disorders (Table 1) [56].

**Table 1:** Neurobiological correlates and overlap of symptoms with other psychiatric disorders [modified from 4].

**disorder**	**symptoms**	**Involved neuroanatomic regions**
**Substance abuse**	Reduction of tension, enhancement of capacity to concentrate in certain situations, emotional stabilization	Striatum, dorsolateral prefrontal cortex, orbitofrontal cortex

**Depressive disorders**	Problems with concentration, lack of drive, feelings of exhaustion, self doubts, social isolation, sleeping problems	Prefrontal cortex, anterior cingulate cortex, hippocampus, amygdala

**Anxiety Disorders**	Self doubts, insecurity, phobic reactions, attention bias	Prefrontal cortex, anterior cinguler cortex, insular- and orbitofrontar cortex, amygdala, ventral striatum, grey layers

**Anti social personality disorder**	Problems abiding to social norms, lower threshold for aggressive-violent behavior lack of adaptive problem solving strategies, low tolerance to frustration	Orbitofrontal cortex, ventromedial prefrontal cortex, limbic system

**Borderline-personality disorder**	Disregulated emotional responsiveness, lack of adaptive problem solving strategies, affective instability, disorder of identity, instable but intensive relationships, inappropriate anger or problems to control anger	Orbitofrontal cortex, dorsolateral and ventromedial prefrontal cortex, amygdala

Due to the comorbidity and deficits caused by ADHD, other impairments often emerge which negatively influence the social and emotional well being of the affected person. Difficulties in the organization of daily duties can lead to problems at work, at home, and in social relationships. Problems with emotion regulation can provoke negative social interactions which further intensify the psychological strain of the affected person and his/her relatives and in turn heighten the risk of developing comorbid disorders. A negative spiral can be detected whose severity differs depending on an individual's characteristics and available resources. The following detailed description of comorbid disorders will underline this.

##### Substance abuse

Prospective studies show an increased rate of substance abuse [7,57]. In many cases a strong association was found between the consumption of substances, behavior disorders in childhood, and a negative social environment [40,50]. Many patients report a better "drive" and ability to concentrate when using stimulating substances. This certainly has consequences for the diagnostic process; first, it has to be clarified if the symptoms can be regarded as reactions to the substance abuse rather than as ADHD specific symptoms [51]. Furthermore, it should be assessed which qualitative sensations result from the consumption of different substances (in particular regarding the reported improvement in attention processing). Results of this assessment will also influence treatment planning.

##### Depressive disorders

Emotional instability and emotional reactivity often occur in adult ADHD. Many patients for example show extreme reactions to frustrating events. Rapid mood changes without apparent reason are also characteristic of the pathology. In one study, a comorbidity of ADHD and major depression was found in 15% of the cases [58]. Furthermore, 7.6% of the sample fulfilled the diagnostic criteria of a dysthymic disorder and 10.4% of a bipolar affective disorder. Hereby it has to be noted that especially the disorder mentioned last includes symptoms similar to those of ADHD, which results in overlapping symptom criteria. Other studies, that regarded ADHD as a risk factor for developing a bipolar affective disorder, attained heterogeneous results. Wilens et al. state that, in their core elements, ADHD and bipolar affective disorders are clearly distinguishable [59].

##### Anxiety disorders

Many ADHD affected persons have developed (dysfunctional) strategies to avoid the confrontation with anxiety-laden situations in order to manage their disorder. But even though an unavoidable confrontation with individual anxiety-laden situations is assumed to have effect on ADHD symptomatology, no causal direction between ADHD and anxiety can be stated [60]. An overall increased level of arousal as well as the tendency to hyper-focus can facilitate the development of an anxiety disorder [61,62]. Different studies about the comorbidity of ADHD and anxiety disorders underline these findings. Biederman reported a life time prevalence rate of comorbid anxiety disorders in 50% of the patients affected by ADHD in adulthood [56].

##### Antisocial personality disorder/delinquency

The presence of oppositional behavior and, consequently, the development of a conduct disorder elevate the risk of developing an antisocial personality disorder [63]. In adults with ADHD, these symptoms are often exhibited in the form of aggressive traffic behavior, delinquency, and as substance and alcohol abuse [64-66]. The domain of delinquency in particular plays a significant role, with different studies highlighting the relation of ADHD, comorbid antisocial personality disorder and delinquent behavior [25,67,68]. In their study on 129 male inmates, Rösler et al. reported a 45% prevalence rate of ADHD, according to the DSM-IV criteria. Hereby, the ADHD subtypes [69] were distributed as follows: 21.7% of the combined type, 21.7% of the predominantly hyperactive-impulsive type and 1.6% of the predominantly inattentive type. With the exception of the last type, all results were significant compared to a control group. Regarding antisocial personality disorder, the authors detected a prevalence rate of 9.3%, whereas among the control group, no person suffered from an antisocial personality disorder. This difference is not statistically significant, but rather reflects a tendency. The strongest relationship between ADHD and antisocial personality disorder was reported for the group of inmates who exhibited conduct disorder [66]. This is of special interest with regard to the frequent comorbid disorders of ADHD in childhood and adolescence [56]. It has to be stated, however, that prevalence rates in numerous other international studies on the interrelation of ADHD and delinquency are lower [66-68].

##### Borderline personality disorder

The comorbidity of ADHD and borderline personality disorder can be regared as the biggest diagnostic challenge. One reason is the substantial overlap of the diagnostic criteria [9,10]. An association of ADHD in childhood and a borderline personality disorder was found in a study of Fossati and colleagues [70]. Among 42 patients with a borderline personality disorder, 59.5% reported ADHD symptoms in childhood. Miller, Nigg and Faraone likewise reported a clear link between ADHD and a borderline personality disorder [71].

## Discussion

### Developmental model of ADHD

**Figure 1:**
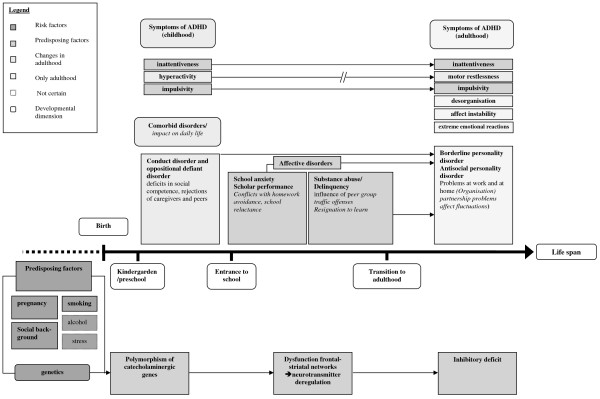
**Developmental psychopathological model of ADHD over the life span [modified from 13]**.

The above cited findings, in association with observations and diagnostic findings from the clinical experience in the treatment of persons affected by ADHD reveal age group specific dysfunctions as well as persistent behavioral factors which result from the neurobiological basis of ADHD (see figure 1). To clarify the aspect of a developmental course across the lifespan, we developed a model. Even though the model is confined in its complexity, it aims at helping the reader to deduce hypotheses about age specific impairments caused by ADHD.

In the developmental model essential features are incorporated that

• heighten the risk to develop ADHD,

• persist along the life span,

• change along the life span, and

• are only valid in adulthood.

Multiple causal predisposing factors form the core of the developmental psychopathology of ADHD. In the aetiopathogenesis of ADHD, the influence of genetic predisposing factors, [17,19] negative prenatal and socio-environmental factors are regarded as certain. Different prospective studies found a relationship between tobacco consumption during pregnancy and ADHD in childhood [72,73]. Besides a generally higher sensitivity to nicotine consumption, the interaction between genetic vulnerability and the smoking behavior of the mother during pregnancy is stressed for an ADHD subtype [74,75]. Other toxins, such as alcohol or drugs, as well as stress are likewise seen as risk factors. However, results from different studies reveal a contradictory picture.

Across the lifespan, the occurrence of ADHD symptoms first peaks during early elementary school age. In this period, most of the ADHD diagnoses are given. Considering the developmental pathway (figure 1) it can be discovered that, with increasing age, the symptom criteria and comorbid disorders from preschool both persist and vary due to the changing social environment of the child. In the school context, skills are often affected which cause deficits in academic performance. Students with ADHD often perform less well compared to students without ADHD [76]. In this context, the comparatively higher comorbidity (compared to students without ADHD) with other learning disabilities (reading or writing disabilities, dyscalculia) must be mentioned [77]. Likewise, school related anxieties can develop which can reach the symptom severity of a phobic disorder [60]. Comorbid affective disorders often emerge, with prevalence rates ranging from 5 to 47% in childhood and adolescence [59,78]. On the one hand these problems can be regarded as a consequence of ADHD, on the other hand they occur due to specific biological mechanisms, such as the linkage with complex dompaminergic gating disturbances; the same brain regions being affected in both disorders (ventral striatum and nucleus accumbens, influenced by the hippocampus and amygdala). Even though the interaction between ADHD, anxiety-, and affective disorders can be regarded as a developmental phenomenon, evidence exists that the co-occurrence of anxiety disorders in children with ADHD seems to increase their risk of developing depressive disorders [79]. As mentioned, it is necessary to note that no causal direction between ADHD, anxiety, and depressive disorders can be stated, but the interrelation of these areas of psychosocial functioning underlines the need for an early intervention [60]. With increasing age, substance abuse and delinquent behavior of individuals affected with ADHD increases [40,51].

At the transition into adulthood, involvement in traffic offences increases. In a prospective study Fischer et al. found a clear relationship between ADHD and rear end collisions, tickets for ruthless driving, driving without a driver's license, suspension of driver's license and driving despite being suspended. Behavior observations while driving revealed a higher incidence of mistakes, which can be seen as a consequence of the impulsive behavior of patients with ADHD. These findings are supported by the results from a driving simulation in which a group of ADHD patients showed slower reaction times with higher variability compared to a control group [47]. Here too, mistakes seemed to be due to their impulsive behavior (e.g. through false reactions). This is in accord with the neurobiological outcomes of various studies. Regarding the developmental model, it can be assumed that along the life span, basic deficits regarding neurotransmitter regulation persist for all age groups comparably. Age specific changes in the disorder become apparent on the behavioral level and are most obvious in adulthood. Regarding the diagnostic criteria, a qualitative change in hyperactivity can be observed which is expressed as motor restlessness (e.g. restlessness of hand and feet or the continuous "playing" with objects like a pen) and/or the experience of „being driven“ (e.g. ADHD affected persons seem to be on the go constantly and/or seem to feel nervous or uncomfortable). Until adulthood, comorbid disorders are subject to continuous development. The characteristics of comorbid disorders, however, are based on the secondary problems that manifested during childhood and adolescence. Different comorbid disorders occur over the lifespan, which is probably due to the changing requirements in adulthood leading to specific impairments in individual areas of functioning (e.g. lower work performance in connection with disorganization). Special attention must be devoted to the already described comorbid borderline personality disorder. The borderline personality disorder in its clinical presentation is very similar to ADHD in adulthood. Studies showed that many affected adults fulfill the criteria of ADHD in childhood. It seems that ADHD in childhood is a risk factor for the development of a borderline personality in adulthood [8,9,71]. A possible relationship between antisocial personality disorder (APS) and ADHD is examined. Among 105 male delinquents with a diagnosis of APS Semiz et al. found a comorbid ADHD in 65% of the cases [80]. Lahey and colleagues detected that the combination of ADHD and a comorbid behavior disorder in childhood can be regarded as a predictor of APS in adulthood [81]. An isolated ADHD, in contrast, did not predict APS in adulthood. It seems that APS in adulthood is caused by a comorbid behavior disorder that manifested itself along the life span, rather than by the ADHD symptomatology. Compared to ADHD in childhood, adults with ADHD are impaired in different areas of functioning (social relationships and partnership due to emotional over-responsiveness, affect instability; occupational area due to disorganization).

## Conclusion

In clinical practice, it is crucial to know during which developmental stage qualitative changes in ADHD occur. In accordance with the current state of knowledge, two points in time seem likely. The preschool age seems to be an important developmental stage in which ADHD symptoms can first be assessed. In many cases, early abnormalities inhibit the full development of a child's resources. The high comorbidity with other psychiatric disorders (e.g. conduct disorder) and the resulting deficits in social competences that often go along with a diminished quality of social contacts must be stressed. At this point in time, preventive steps should be taken to counteract the negative effects of ADHD.

The transition into adulthood can be regarded as a second crucial developmental transition point. The assumption that ADHD is a disorder that only occurs in childhood has dominated clinical psychology for many years. Due to the high comorbidity with other disorders, ADHD symptoms are often overlooked; they do, however, seem to play an important role in the manifestation of other comorbid disorders. This complicates the diagnostic process because symptoms of ADHD and comorbid disorders can overlap. Affective disorders, as for example the borderline personality disorder and the antisocial personality disorder, can be mentioned here. For this reason and for the diagnostic assessment in clinical practice, the developmental aspect of ADHD is fundamental. Evidence for childhood ADHD is a diagnostic criterion for ADHD in adulthood and knowledge of the developmental course improves possibilities for a comprehensive intervention.

To expand our knowledge one could ask a second question, namely what causes the differences between symptom manifestation of ADHD in childhood and adulthood? According to neuropsychological findings, the same neurobiological model underlies ADHD over the whole life course. The symptomatological differences between ADHD in childhood and adulthood seem to be caused by environmental and social interaction factors. As mentioned already, ADHD symptoms begin to occur during preschool and elementary school age. These symptoms are more clearly circumscribed than is the case in adulthood. Consequently, it can be assumed that over the developmental course, on the one hand more areas of functioning will be negatively affected by ADHD (which is the case at the transition to adulthood), and on the other hand a higher comorbidity can be found. Here again, the relation with the secondary disorder becomes apparent [63], which highlights the need for a preventive and, if already manifested, early therapeutic intervention.

## Abbreviations

ADHD: Attention-Deficit/Hyperactivity Disorder; APS: Antisocial Personality Disorder; fMRI: functional Magnetic Resonance Imaging.

## Competing interests

The authors declare that they have no competing interests.

## Authors' contributions

SC and FP were equally responsible for defining the research question. FP was responsible for the description of the developmental course of ADHD in childhood and adolescence. SC was responsible for the phenomenology of adult ADHD and the realization of the developmental model. Both authors read and approved the final version.

## Pre-publication history

The pre-publication history for this paper can be accessed here:


